# Nuclear factor erythroid 2-related factor 2 modulates HER4 receptor in ovarian cancer cells to influence their sensitivity to tyrosine kinase inhibitors

**DOI:** 10.37349/etat.2021.00040

**Published:** 2021-04-30

**Authors:** Ibrahim H. Kankia, Poornima Paramasivan, Matthew Elcombe, Simon P. Langdon, Yusuf Y. Deeni

**Affiliations:** 1 Division of Health Sciences, School of Applied Sciences, Abertay University, Dundee DD1 1HG, UK; 2 Cancer Research UK Edinburgh Centre and Edinburgh Pathology, Institute of Genetics and Cancer, University of Edinburgh, Edinburgh EH4 2XU, UK; 3 Department of Biochemistry, Faculty of Natural and Applied Sciences, Umaru Musa Yar’adua University, Katsina PMB 2218, Nigeria; 4 Department of Microbiology and Biotechnology, Faculty of Science, Federal University Dutse, Dutse PMB 7156, Nigeria; Agostino Gemelli University Policlinic, Italy

**Keywords:** Cancer, ovarian, nuclear factor erythroid 2-related factor 2, HER4-receptor, regulation, rexinoid, erlotinib, lapatinib

## Abstract

**Aim:**

Nuclear factor erythroid 2-related factor 2 (NRF2) is a key component in the cell’s response to oxidative and electrophilic stress and is a transcription factor regulating the expression of a collection of anti-oxidative and cytoprotective genes. Human epidermal growth factor receptor 4 (HER4/erbB4) regulates growth and differentiation in many cancer types. Here, NRF2 and HER4 receptor interactions were investigated in a panel of ovarian cancer cell lines.

**Methods:**

Pharmacological [tert-butylhydroquinone (tBHQ) and retinoid/rexinoid, bexarotene] and genetic [small interfering RNA (siRNA)] manipulations were used to activate or inhibit NRF2 function in the cell line panel (PE01, OVCAR3, SKOV3). Activity of the HER-targeted tyrosine kinase inhibitors, erlotinib (ERL) and lapatinib (LAP), was evaluated after NRF2 activation.

**Results:**

While tBHQ increased the levels of both phosphorylated-NRF2 (pNRF2) and HER4 in PE01, OVCAR3 and SKOV3 cells, bexatorene and NRF2-target siRNA treatment decreased pNRF2 and total HER4 levels. The tBHQ-dependent pharmacological activation of NRF2 attenuated the therapeutic effectiveness of ERL and LAP. Analyses of gene expression data from a HER4 driven reporter system and *in vitro* or *in vivo* cancer models, support NRF2 regulation of HER4 expression.

**Conclusions:**

These results support the presence of signaling interaction between the NRF2 and HER4 receptor pathways and suggest that intervention modulating this cross-talk could have anticancer therapeutic value.

## Introduction

Nuclear factor erythroid 2-related factor 2 (NRF2) is a basic leucine zipper protein that regulates the cell’s defense to oxidative stress by activing transcription of batteries of anti-oxidant genes [1–3]. NRF2 is a master regulator of multiple genes and a key factor for cytoprotection including anti-tumour effects, neuroprotection and anti-inflammatory response [1, 4, 5]. Located in the cytoplasm, NRF2 degrades under normal and unstressed conditions, but in the presence of oxidative stress, it moves to the nucleus where it dimerises with macrophage activating factor (Maf) proteins, then binds to antioxidant response elements (AREs) and initiates transcription of anti-oxidative genes [1, 3, 6, 7]. Previously identified activators of NRF2 include tert-butylhydroquinone (tBHQ) [8] while inhibitors include the retinoid bexarotene [9, 10].

The human epidermal growth factor receptor 4 (HER4/erbB4) is a cell surface receptor and member of the epidermal growth factor receptor (EGFR) tyrosine kinase family. This receptor family play major roles in cancer growth and progression [11–14]. HER4 was initially thought to be specific for the regulation of development, maintenance, function and behaviour of the central nervous system [15–21]. However, many studies have identified HER4 to be relevant to carcinogenesis, cancer treatment and prognosis [22–32]. In ovarian cancer, high levels of HER4 have been associated with chemoresistance in both cell line models [33] and in patient samples where it has been associated with reduced overall survival [34]. HER4 can be expressed as alternatively spliced isoforms in ovarian cancer cells [33] and expression of a specific HER4 isoform, cytoplasmic (CYT)-1, has been associated with both increased cell growth and poor survival [35]. HER4 is activated by neuregulins, e.g., heregulin (HRG, H), whereupon it can either homodimerise or heterodimerise with any other member (EGFR, HER2 or HER3) of the HER receptor family.

NRF2 has been shown to be aberrantly activated in many ovarian cancers and this is frequently associated with a loss of its inhibitory complex [36]. Overexpression of NRF2 is also found in many ovarian cancers [36–38]. NRF2 could have a direct link with HER4 via signalling pathways that include phosphatidylinositol 3-kinase (PI3K)/serine-threonine kinase (AKT)/mammalian target of rapamycin (mTOR), mitogen-activated protein kinase (MAPK), and signal transducers and activators of transcription (STAT) [1, 31, 39–41]. Previous studies in ovarian cancer cells have reported on the transcriptional regulation of HER2 and HER3 by NRF2 and showed associations between NRF2 function, HER2/HER3 signalling, reactive oxygen species (ROS) generation and glutathione depletion [40, 41]. Furthermore, Kankia et al. [10] demonstrated NRF2 regulation of HER1 expression and the modulatory effect of NRF2 on the sensitisation of ovarian cancer cells to receptor tyrosine kinases inhibitors (RTKi) and anticancer drugs targeting HER1/HER2 receptors.

In this study, we explored the cross-talk between NRF2 and HER4 and the potential contribution of the HER4-NRF2 axis in influencing the cellular response to lapatinib (LAP) and erlotinib (ERL).

## Materials and methods

### Cell culture

The PEO1, SKOV3 and OVCAR3 cell lines were cultured at 37°C in 5% CO_2_: 95% air in RPMI 1640 medium containing fetal calf serum (FCS, 9:1) together with glutamine (2 mM), sodium pyruvate (1 mM), streptomycin (100 μg/mL) and penicillin (100 U/mL). For experiments, cells were grown for 24 h in RPMI 1640 medium with 5% double charcoal stripped fetal bovine serum (FBS, Fisher) replacing FBS. HRG-β1 (Sigma) was prepared as a 1 μM solution containing 5% trehalose and 10% FCS in phosphate buffered saline (PBS) and diluted to 1 nM with media within experiments. tBHQ (Sigma) and bexarotene (Carbosynth) stock solutions were prepared in dimethyl sulfoxide (Fisher).

### Cloning of *HER4* promoter

The *HER4* promoter was isolated and cloned using a previously described method [40, 41]. The primer sequences used were *HER4* forward: 5-CCGCTCGAGGAGTGGGAAATGG-AGATCAAGGTC-3′ and *HER4* reverse: 5′-GGACAAGTGTGA GGAAAGC-TGAGAGCCATGGCATG-3. Human DNA was extracted from cells using the DNeasy Blood and Tissue Kit (Qiagen). Genomic DNA (100 ng) was used to amplify the HER4 promoter sequences and real-time polymerase chain reaction (RT-PCR) conditions using MyFi mix (Bioline) included denaturation at 95°C for 7 min followed by 35 cycles at 95°C for 30 s for denaturation, 50°C for 30 s for annealing, and 72°C for 90 s for extension with a final extension for 10 min at 72°C. The created PCR products were then run and extracted from an agarose gel (Qiagen), followed by digestion using XhoI and HindIII restriction enzymes (Promega), then ligation into pGL3 vector (Promega) to create a *HER4* promoter construct (prHER4) driving the expression of luciferase gene which was then used in dual luciferase reporter assays (Promega). The cloned sequence was authenticated by sequencing (http://www.dnaseq.co.uk/) the plasmids. Lipofectamine 3000 (Life Technologies) was used to transfect the cloned plasmid into cell lines.

### Luciferase reporter assay

To analyse HER4 transcriptional regulation, the *HER4* promoter sequence was transfected into target cell lines. Cells (2 × 10^5^ cells per well) were plated in triplicate wells for 18 h, then transfected (using Lipofectamine 3000) with 1 mg of pGL3 basic vector (Promega) with or without cloned fragments of *HER4* promoter. pRL-CMV vector (Promega, 0.2 μg) was also co-transfected to serve as a measure of transfection efficiency. After 24 h, cells were treated and protein lysates transferred to opaque white bottom 96-well plates. The firefly luciferase (from the cloned promoter) and Renilla luciferase (from the internal control) in the harvested lysates were then measured according to the manufacturer’s protocol (Promega) with luminescence being measured in a luminometer (MODULUS, Promega).

### Cytotoxicity assay

Cell viability was assessed with the CellTiter-Glo^®^ 2.0 assay kit (Promega). Cells were plated in 96-well plates and left for 24 h. After treatment with drugs as described, the plates were left for 30 min at room temperature. CellTiter-Glo 2.0 reagent was added at a volume equal to that of medium present in each well and cell lysis was induced by placing the plate on an orbital shaker and then incubating at room temperature for 10 min to stabilize the luminescent signal read by luminometer (MODULUS, Promega). The assay measures ATP which in turn indicates the level of live and metabolically active cells.

### Western blot analysis

Cells were plated into 60 mm cell culture plates and grown until 70% confluence. After treatment, cells were trypsinized, washed with PBS. Protein lysates were prepared using Radio-Immunoprecipitation Assay (RIPA) buffer (Pierce Biotech) together with protease and phosphatase inhibitor cocktail (Pierce Biotech) and sonicated for 2 cycles of 10 s. After shaking on ice for 15 min, the lysates were centrifuged (14,000 g for 15 min) and supernatant collected. The protein content was measured by Bradford assay (Sigma-Aldrich) using bovine serum albumin as reference. Lysates were added to sample loading buffer [NuPAGE lithium dodecyl sulfate (LDS), Invitrogen], heat denatured (70°C for 10 min) and stored at –20°C until used. Lysates were loaded onto Nupage Bis-Tris gels (Life Technologies) and electrophoresed at 200 V for 1-2 h. Proteins were then transferred onto polyvinylidene fluoride (PVDF) membranes (GE Amersham) within the Invitrogen™ iBlot™ 2 Dry Blotting System. Membranes were first blocked before being incubated with primary antibodies (either for 2 h at room temperature or overnight at 4°C). Primary antibodies targeted the following: HER4 (Rabbit; Abcam, Ab32375), phosphorylated-NRF2 (pNRF2; Rabbit; Abcam, Ab76026), phospho-AKT (pAkt)-Ser473 (Rabbit; Cell Signaling, Ab9271) and b-actin (Rabbit; Abcam, 801). Primary antibody incubation was followed by incubation with horseradish peroxidase (HRP)-linked secondary (anti-Rabbit; Cell Signaling, 7,074; 30 min at room temperature). Pierce enhanced chemiluminescence (ECL) 2 western blotting substrate (ThermoScientific) and reagents were then used according to manufacturer’s protocol. Blots were analyzed using a Syngene G-BOX Chemi-XX6 Gel Documentation System (Synoptics, UK). Densitometry was measured using Image J software and integrated optical densitometry analysis of each band was then performed. β-actin was used as loading control and the values shown are normalized against this.

### Small interfering RNA (siRNA) knockdown studies

A specific siRNA (Hs_NFE2L2_6, Qiagen) was used to knockdown NRF2. Cells were plated in triplicate either in 24-well plates (0.5 × 10^5^ cells) or in 60 mm plates with cells grown on poly-L lysine coated coverslips (0.5 × 10^6^ cells). After 24 h, cells were then co-transfected using either 20 pmol siRNA and 1 μg of different pGL3 promoter constructs (for 24-well plates) or 75 pmol siRNA only (for 60 mm plates) and incubated for a further 24 h. Cells transfected in 24-well plates were used for the dual luciferase assay while those in 60 mm plates were harvested for immunoblotting. In all experiments, scrambled siRNA of equivalent quantity to the NRF2-siRNA was used as a control. All transfections were used using Lipofectamine 3000 (Life Technologies).

### Determination of ROS and glutathione levels

For measurement of ROS, cells (2 × 10^4^/mL) were plated in triplicate in opaque 96-well plates in phenol red-free medium (100 mL). After 18 h, an equal volume (100 mL) of a 2’,7’-dichlorofluorescein diacetate (DCFDA, 50 mM) solution was added to each well and left to react for 45 min at 37°C. ROS levels were measured by fluorescence using excitation (485 nm) and emission (535 nm) spectra in a multiplate reader (MODULUS, Promega). For normalization, cells were stained with Coomassie blue stain (Sigma) for 1 h then washed with distilled H_2_O. To release the dye, sodium dodecyl sulphate (10% solution) was added with 10 min shaking. Absorbance was read at 595 nm in the above multiplate reader.

For measurement of glutathione, cells (1 × 10^4^/mL) were plated in white clear bottom 96-well plates and left for 18 h. Media were removed from the wells. The glutathione (GSH)/GSSG-Glo™ Assay (Promega) was used according to the manufacturer’s protocol. Total glutathione lysis reagent (50 mL) was added to each well and lysates were transferred to a 96-well opaque plate. Plates were shaken at room temperature for 5 min. Luciferin Generation Reagent (50 mL) was added to each well and plates shaken and left at room temperature for 30 min. Luciferin Detection Reagent (100 mL) was added and plates were shaken for 15 min. Luminescence was then measured using a luminometer (MODULUS, Promega).

### Bioinformatic methods

Microarray data from the Gene Expression Omnibus (GEO, https://www.ncbi.nlm.nih.gov/geo/) repository was mined to retrieve gene expression data in response to ERL and LAP treatments. Datasets obtained from GEO were GSE116445, GSE116442 [42]; GSE33072 [43]; GSE80316 [44]; GSE67051 [45]. The statistical analysis of the gene expression variances obtained was undertaken using the GEO’s analytical tool, GEO2R, that utilizes R programming and Bioconductor packages, from which the fold change of gene expression was calculated and then heat maps were created.

### Statistical analysis

Statistical analyses were performed using Graph pad. The significance values of the differences of pooled results were determined by either independent-tests or one-way analysis of variance (ANOVA) followed by post hoc Tukey’s tests. Significance was defined as *: *P* < 0.05, **: *P* < 0.01, ***: *P* < 0.001 and ****: *P* < 0.0001.

## Results

### Activation of NRF2 increases expression of HER4

**Figure 1.**
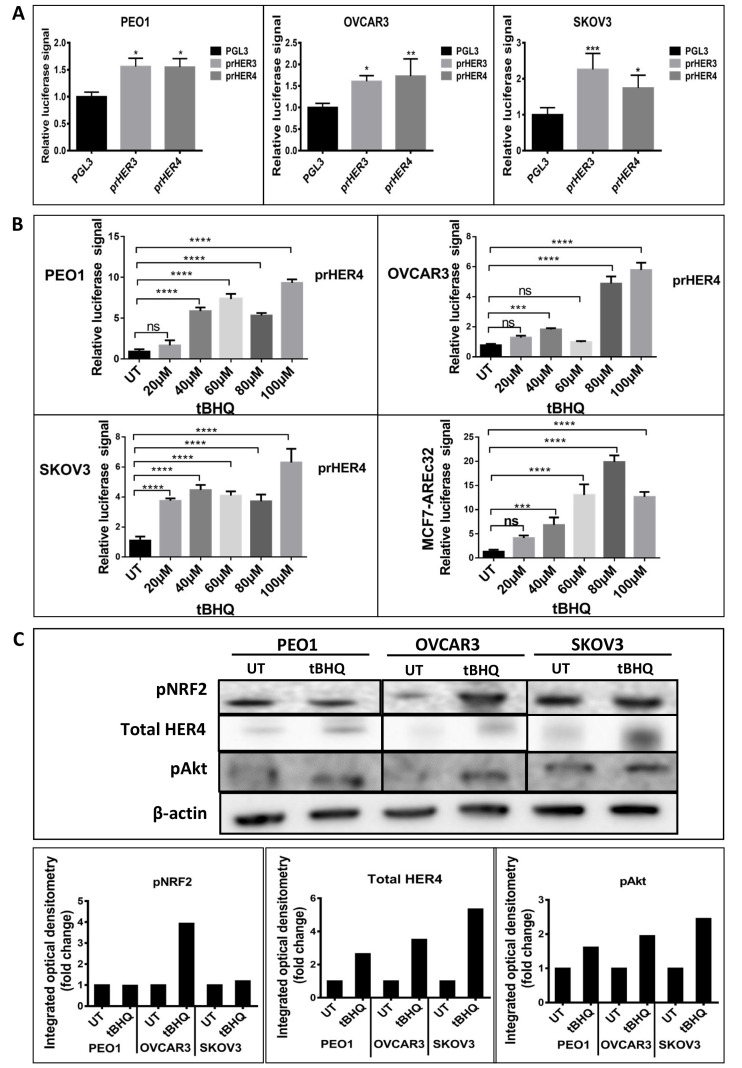
NRF2 regulation of HER4 expression. (A) Ovarian cancer cell lines demonstrate basal transcription of HER3 and HER4 respectively. Cell lines were transfected with either control pGL3 vector or pGL3 vector with fragments of either *HER3* (prHER3) or *HER4* (prHER4) promoter driving expression of luciferase. Data shown are mean of triplicates normalized to the value of pGL3 [ANOVA followed by Tukey’s post hoc test (*: *P* <0.05, **: *P* < 0.01, ***: *P* < 0.001)]; (B) tBHQ treatment increases transcription of HER4 in a dose dependent manner. MCF7-AREc32 contains stably cloned 8 × *cis*-ARE driving NRF2 dependent expression of luciferase gene [8]. Means ± standard deviation (SD) of triplicates normalized to the value of untreated (UT) are shown [ANOVA followed by Tukey’s post hoc test (***: *P* < 0.001 and ****: *P* < 0.0001; ns = non-significant)]; (C) western analysis following 24 h treatment with tBHQ (100 μM) indicated increased HER4 and pAkt expression in all cell lines. Bar charts indicate fold-changes of expression in tBHQ treated cells *versus* UT cell

We first investigated whether HER4 expression could be regulated by NRF2. To achieve this, we created a luciferase transcriptional reporter construct using the proximal promoter region of *HER4* and evaluated the transcriptional expression in a group of ovarian cancer (PEO1, OVCAR3 and SKOV3) cell lines. The comparison was made with HER3, which has been shown to be regulated by NRF2 [40]. All three cell lines demonstrated an increased basal level of luciferase activity (Figure 1A). Upon addition of the NRF2 activator, tBHQ, there was a concentration-dependent increase in *HER4* promoter driven transcriptional activity in all cell lines. A similar pattern of response to tBHQ was observed in an MCF7 breast cancer cell line AREc32 [8] stably transfected with *cis*-elements of the antioxidant response (Figure 1B). To assess protein levels, western blot analysis was used to demonstrate that tBHQ (100 μM) treatment produced increased levels of pAkt, pNRF2 and total HER4 levels, which was most evident in OVCAR3 cells and less obvious in PEO1 and SKOV3 cell lines (Figure 1C). These results suggest that phosphorylation and activation of NRF2 protein caused upregulation of HER4 levels. Taken together, these observations imply that the NRF2 and HER4 pathways might be subject to co-regulatory mechanisms partly mediated by pAkt.

### Inhibition of NRF2 causes downregulation of HER4 expression

**Figure 2.**
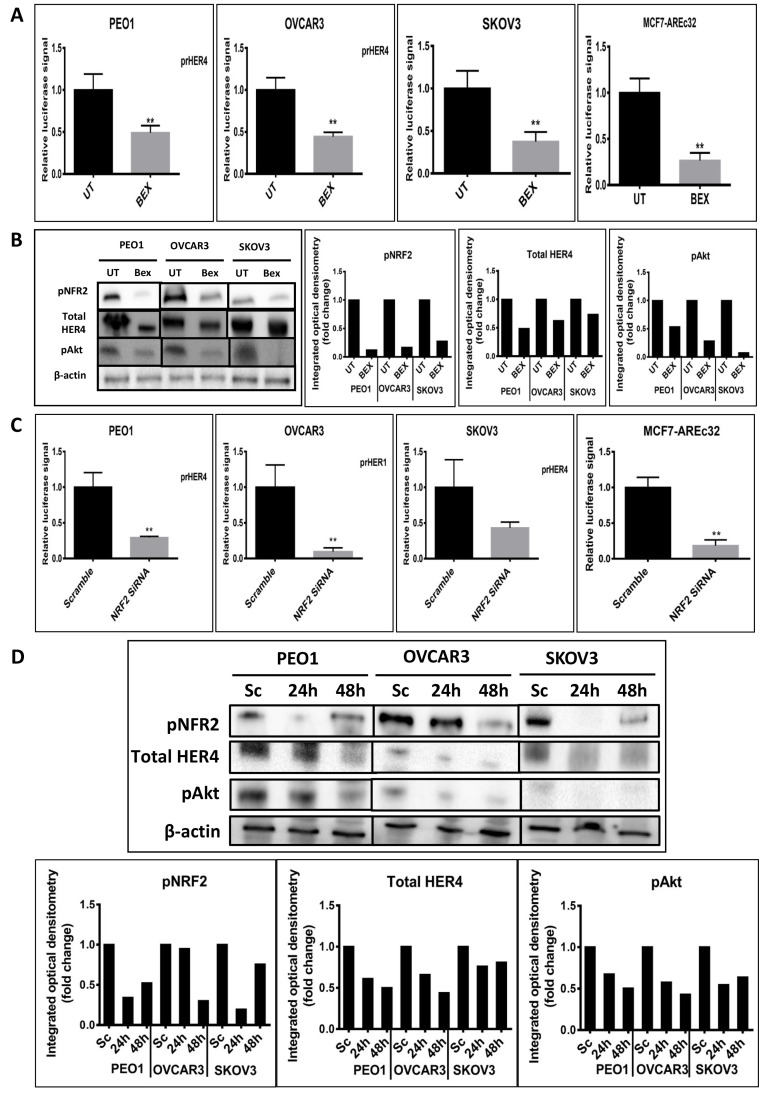
Inhibition of NRF2 by bexarotene or siRNA is associated with downregulation of HER4. (A) Luciferase assay demonstrating downregulation of HER4 by bexarotene in cell lines. Cells were transfected with either pGL3 basic vector or pGL3 basic vector with cloned NRF2 AREs driving the expression of luciferase gene. After 24 h, cells were either UT or treated with 2.5 μM bexarotene for a further 24 h. Lysates were prepared and luciferase activity was measured as described in Materials and methods; (B) western analysis after treatment with bexarotene indicated decreased expression of HER4, pNRF2 and pAkt. Cells were either UT or treated with 2.5 μM bexarotene for 24 h before being processed for western analysis. Signal quantitation is shown in the bar charts; (C) siRNA-reduction of NRF2 decreases transcription of HER4. Cells were treated with NRF2-targeted siRNA or scrambled siRNA as described in Materials and methods; (D) western blot analysis after siRNA-mediated knockdown of NRF2 demonstrated expression of HER4 receptor, pNRF2 and pAkt proteins in cell lines. Cells were either transfected with scrambled siRNA (Sc) or transfected with 75 pmol of NRF2 siRNA (Si). After 24 h and 48 h, cells were processed for western analysis using indicated antibodies. β-actin was used as loading control. The bar chart indicates the expression levels as fold change. Data shown in figures (A) and (C) are the mean ± SD of triplicates, normalized to untreated or scramble control expressed as fold change with statistical significance determined by student’s *t*-test (*: *P* < 0.05, **: *P* < 0.01 and ***: *P* < 0.001)

Since HER4 protein induction by tBHQ seemed likely a result of transcriptional upregulation, experiments were next undertaken to inhibit NRF2 with the expectation that HER4 might be down regulated. We examined the transcriptional level of HER4 and then the protein levels of the HER4 receptor, pNRF2 and their downstream substrate, pAkt following the inhibition of NRF2 function by bexarotene or with siRNA (Figure 2). To achieve this, the transcriptional reporter assay for HER4 receptor was used. The ovarian PEO1, OVCAR3 and SKOV3 cancer cell lines were individually transfected with the reporter systems and either treated with 2.5 μM bexarotene for 24 h or cotransfected with 75 pmol NRF2 specific siRNA for 24 h and 48 h. The results indicated transcriptional downregulation of HER4 and significant repression of HER4 and decreases in both pAkt and pNRF2 levels in all 3 cell lines. The findings indicate that inhibition of NRF2 function was linked to inhibition of the HER4 pathway. This is consistent with the antioxidant response and HER4 pathways being co-regulated and with NRF2 regulating HER4 expression and protein levels.

### tBHQ activation of NRF2 enhances cellular proliferation and survival of ovarian cancer and attenuated the cytotoxic and anticancer effects of LAP and ERL

**Figure 3.**
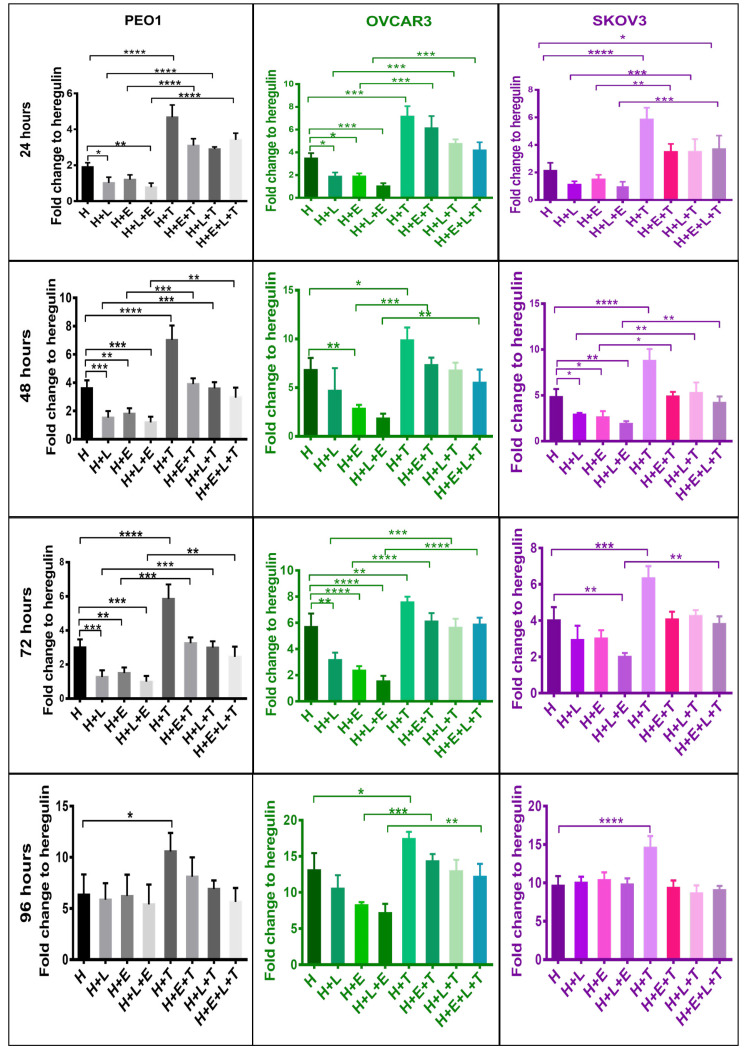
NRF2 activation confers cytoprotection against HER family targeting drugs, LAP (L) and ERL (E) in ovarian cancer cell lines. Cells were plated in media containing 1 nM H or treated with media containing 1 nM H and 5 μM of HER family kinase inhibitors: LAP (H + L), or ERL (H + E), or LAP and ERL (H + L + E), or 100 μM tBHQ (H + T), or tBHQ and LAP (H + L + T), or tBHQ and ERL (H + E + T) or combination of both inhibitors and tBHQ (H + E + L + T). tBHQ (100 μM) was added 5 h in advance and prior to treatments with inhibitors. Cell survival number was assessed indirectly and relative to control by use of the CellTiter-Glo assay (Promega). Values shown are means ± SD of triplicates adjusted relative to control. Statistical significance was calculated between H + L, H + T, and H + L + T groups by One-way ANOVA followed by Tukey’s post hoc test according to the scale (*: *P* < 0.05, **: *P* < 0.01, ***: *P* < 0.001, and ****: *P* < 0.0001)

**Figure 4.**
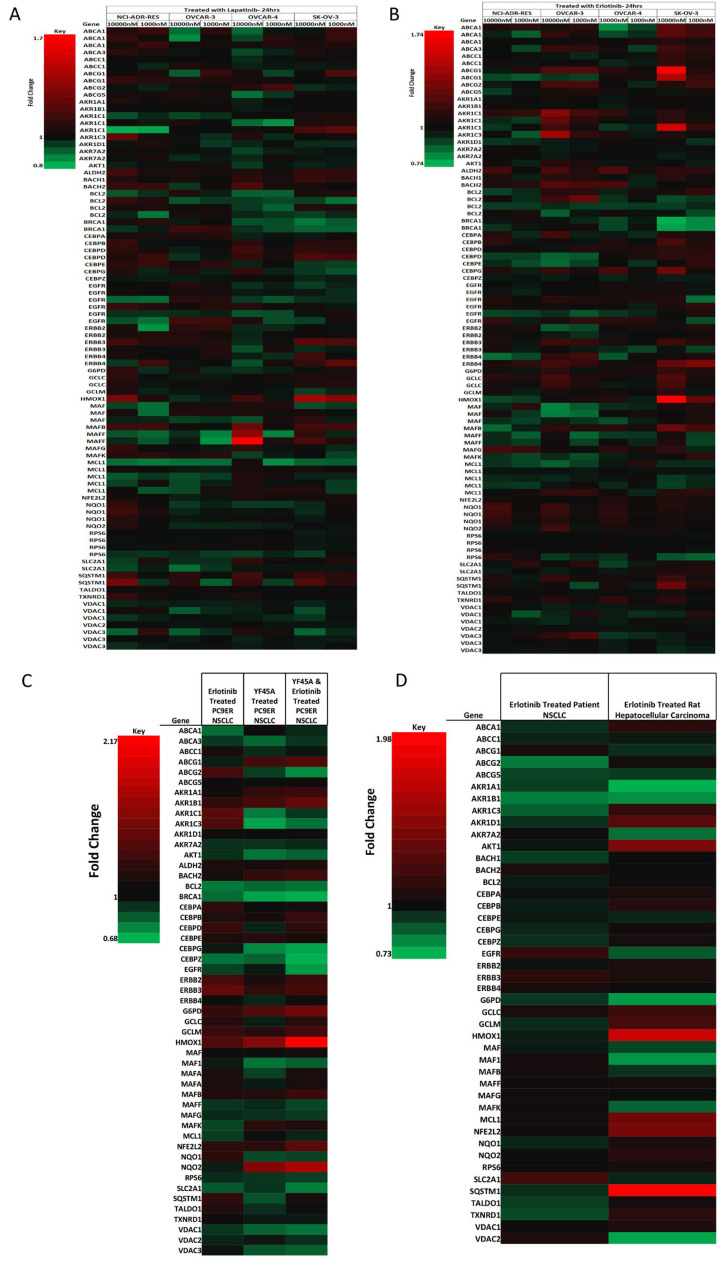
NRF2 network dependent molecular responses to LAP or ERL. Heatmap showing fold-change differential expression of genes within the NRF2 network relative to control treatment of a panel of ovarian cancer cells (NCI-ADR-RES, OVCAR3, OVCAR4, and SKOV3) treated for 24 h with (A) LAP (1 μM and 10 μM); (B) ERL [1 μM and 10 μM, significance analysis of microarrays (SAM) false discovery rate (FDR) = 10%] [43]; (C) heatmap showing fold-change differential expression of genes within the NRF2 network relative to control treatment of non-small cell lung cancer (NSCLC) cell lines (PC9ER) treated for 12 h with ERL (5 μM) or YF45A (0.2 μM) alone, or combination of ERL and YF45A (SAM FDR = 10%) [44]; (D) heatmap showing fold-change differential expression of genes within the NRF2 network relative to control treatment of *in vivo* NSCLC patients and rat hepatocellular carcinoma xenograft tumor with ERL (SAM FDR = 10%) [45]. Red represents increased expression and green decreased expression relative to the median of the controls

Several studies have shown NRF2 to increase resistance to chemotherapeutic agents and enhance the cellular proliferation of cancer cells [8, 46–49]. Moreover, several HER-targeted tyrosine kinase inhibitors (TKIs) including LAP and ERL are used to treat HER family overexpressing (or mutant) cancers and are being evaluated against ovarian cancer [50–53]. In this part of the study the ovarian cancer cell lines, which are known to express HER family receptors and NRF2, were firstly examined for whether activation of NRF2 would modulate cellular response to LAP and ERL. The cells were grown in media containing 5% charcoal-stripped FBS and 1 nM HRG and then treatments were undertaken. The results first showed that NRF2 activation by tBHQ alone enhanced proliferation of these cell lines (Figure 3). However, exposure of these cells to the HER receptor inhibitors, LAP and ERL, inhibited the proliferation of all the cell lines for up to 72 h of treatment, but with a loss of the growth inhibitory and cytotoxic effect at 96 h. Generally, LAP appeared to be a more effective cytotoxic and a better inducer of NRF2, HER family receptors and NRF2-dependent genes (Figures 3 and 4). Pretreatment of cells with 100 μM tBHQ for 5 h prior to addition of LAP and ERL significantly protected cells from the growth inhibitory actions of these drugs. This was observed in all 3 cell lines at all treatment time points examined. These results (Figures 1–3) suggest that NRF2 activation led to cellular protection, induction of HER4 and perhaps eventual resistance to LAP and ERL whose actions are specific to inhibition of HER family receptor function. Furthermore, these findings provide support for a direct link and interaction between HER family receptor expression and NRF2 antioxidant response pathway.

### Genetic reprogramming and NRF2 signalling following ERL and LAP treatment

To further support and confirm our findings, we retrieved and mined published microarray data of *in vitro* and *in vivo* gene expression studies in ovarian, lung and liver cancer models that have been treated with LAP or ERL [42–45]. First, we examined and analyzed the gene expression data on the response of ovarian cancer cells NCI-ADR-RES, OVACR-3, OVACR-4 and SK-OV-3 to ERL and LAP treatment [42], particularly in the context of dynamic changes in the NRF2 network and signalling pathway. We identified significant changes relative to control in the expression of genes within the NRF2 network following LAP or ERL therapy. Many of the differentially expressed genes in the NRF2 network [54–57] appeared to be modulated by ERL or LAP treatment at low (100 nM) or high (1 μM) concentrations. Selected changes in the NRF2 network are shown in the heatmaps (Figure 4A and B). The higher concentration of LAP or ERL used produced greater effects on gene expression changes in ascending order of magnitude in terms of down- and up-regulation of genes. Following ERL or LAP treatment, genes within the NRF2 network that markedly changed were transport, detoxification and metabolism related ABCA1, ABCA3, ABCC1, ABCG1, ABCG2, ABCG5, AKR1A1, AKR1B1, AKR1C1, AKR1C3, AKR1D1, AKR7A2, G6PD, GCLC, GCLM, HMOX1, NQO1, NQO2, SLC2A1, SQSTM1, TALDO1, TXNRD1, VDAC1 and VDAC2; DNA damage and repair related BRCA1; stability, transcription and epigenetic control related BACH1, BACH2, Maf, Maf1, MafB, MafF, MafG, MafK; cell signaling, proliferation, inflammation and immunity related, AKT1, CEBPA, CEBPB, CEBPE, CEBPG, CEBPZ, ERBB1, ERBB2, ERBB3, ERBB4 and RPS6; cell cycle regulation, cell survival and death related BCL2 and MCL1. Interestingly, the expression of ERBB4 (HER4) correlates with the expression of NRF2 (NFE2L2) levels in all the cell lines when treated with either ERL or LAP. For all the cell lines used for the study, NRF2 expression appeared to be drug dose dependent and the expression of HER4 follows the NRF2 expression trend, as well as that of AKT and heme oxygenase-1 (HO-1, a well known classical NRF2-dependent gene).

To check whether NRF2 and HER4 follow the same expression pattern in different cell lines and cancer models, we retrieved and analyzed the microarray gene expression data and profiles of ERL resistant NSCLC PC9 cells [44] treated with ERL or histone deacetylase (HDAC) inhibitor YF454A alone or their combination (Figure 4C). The combination of ERL and HDAC inhibitor YF454A was shown to be a more effective anticancer therapy against NSCLC with EGFR-TKI resistance [44]. Interestingly we could observe that NRF2 and HER4 expression followed a similar pattern and the same order as with the ovarian cancer line models. In addition, this analysis provided further evidence for the contribution of epigenetics to the distinct mechanisms of alterations of NRF2 function by the HDAC inhibitor [41] and concomitant upregulation of the NRF2 levels in this model. Combination treatment with ERL and YF45A caused NRF2 levels to be downregulated more than with the individual/single treatments. Further, analysis of ERL treated rat hepatocellular carcinoma and NSCLC patient tissue microarray data [43, 45] confirmed our observed perturbations in NRF2 network by exhibiting similar trend patterns and concomitant directional expression of NRF2 and HER4 (Figure 4D) as with the ovarian (Figure 4A and B) and lung (Figure 4C) cancer cell lines. This supports and strengthens the notion that NRF2 and HER4 follow the analogous expression in ovarian, lung and liver cancer models, and plausibly NRF2 regulates HER4 expression in all these cells/tissues. This regulation of HER4 expression by NRF2 appeared to be modulated by LAP or ERL drug, which may also be a contributing determinant and basis of cellular sensitivity and response (susceptibility and resistance) of some cancers to LAP and/or ERL.

### Acquired cross-resistance to LAP and increased levels of HER4, pNRF2, pAkt and NRF2-dependent HO-1 in ERL resistant PEO1 (PEO1/ERL) ovarian cancer cell line

**Figure 5.**
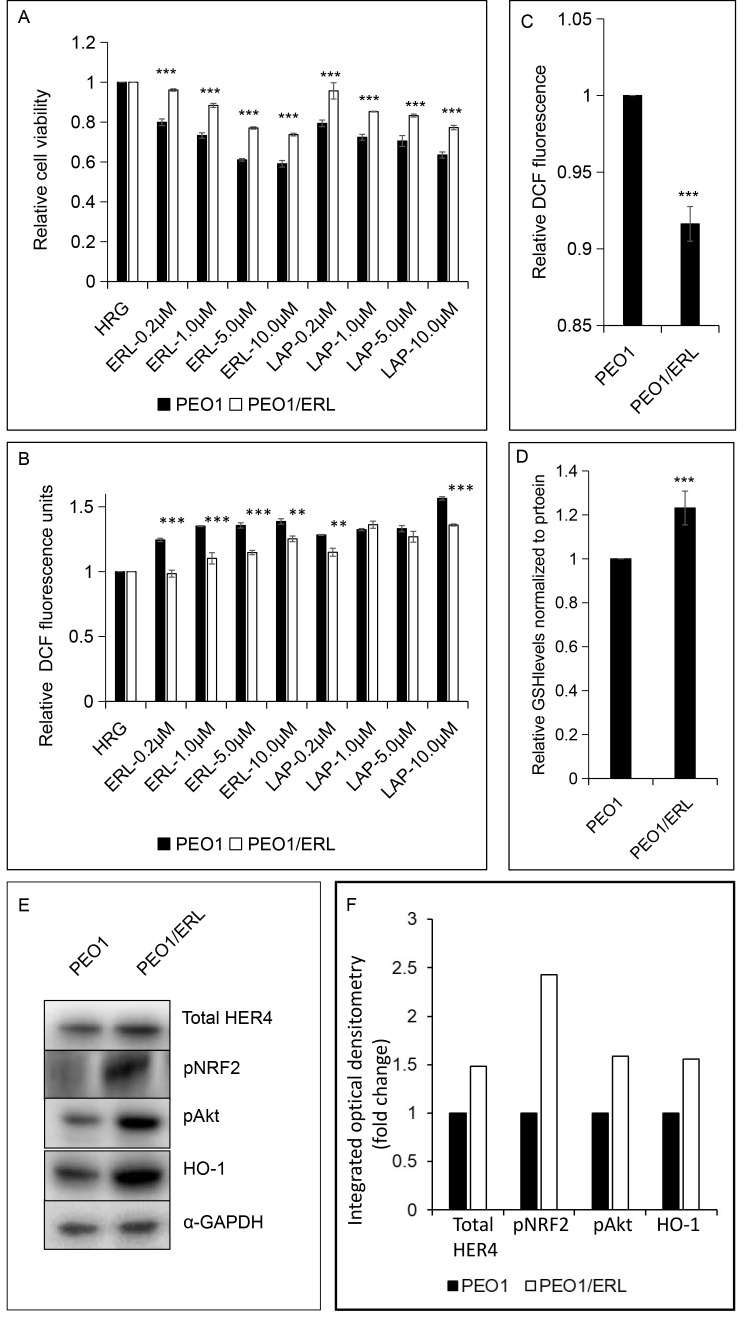
ERL resistant PEO1 (PEO1/ERL) ovarian cancer cell line showing (A and B) increased survival to ERL and cross-resistance to LAP; (C and D) decreased basal ROS and increased basal GSH levels; and (E and F) increased basal HER4, pNRF2, pAkt and NRF2-dependent HO-1 levels relative to the parental PEO1 cell line. The bar charts (A-D) indicate the expression levels as fold change. Data shown are the mean ± SD of triplicates, normalized to control and expressed as fold change with statistical significance determined by student’s *t*-test (**: *P* < 0.01 and ***: *P* < 0.001)

To further explore the concept that NRF2 status and function in the cell could be a contributing determinant and basis of HER4 receptor expression and cellular sensitivity and response of some cancers to LAP and/or ERL, we developed an ERL resistant PEO1 cell line using a previously described approach [43–45]. The mechanism of growth inhibition of ERL and LAP (Figure 5A) towards both the parental PEO1 and ERL resistant PEO1/ERL ovarian cancer cell lines appeared to be associated with ROS generation (Figure 5A and B). The newly established ERL resistant PEO1/ERL cell line had a decreased constitutive level of ROS (Figure 5C) and an increased constitutive total GSH level (Figure 5D) and had acquired cross-resistance to LAP (Figure 5A). Interestingly, the ERL resistant PEO1/ERL cells have increased levels of HER4 (1.5-fold), pNRF2 (2.5-fold), pAkt (1.5-fold) and NRF2-dependent HO-1 (1.5-fold) relative to the parental PEO1 ovarian cancer cells (Figure 5E and F). This is consistent with the suggestion that NRF2 activation led to induction of HER4, cellular protection, and perhaps eventual resistance to ERL and LAP. Thus, these observations support the assertion that HER4 expression is regulated by NRF2 and may also be a contributing determinant and basis of cellular sensitivity and response (susceptibility and resistance) of some cancers to ERL and perhaps to LAP.

## Discussion

In this study, we have demonstrated that the HER4 receptor is regulated by NRF2. NRF2 activation by tBHQ was associated with increased levels of HER4 protein while inhibition of NRF2 with the retinoid bexarotene or use of NRF2 targeted siRNA decreased HER4 expression Previous studies have demonstrated that NRF2 transcriptionally regulates HER1, HER2 and HER3 receptors [40, 41] and this present study now indicates that HER4 is also regulated. Pharmacological activation of NRF2 with tBHQ upregulated HER4 both at the transcriptional and protein levels. tBHQ is a known effector and activator of NRF2 [8]. The upregulation of HER4 at protein level is concomitant with increased expression of pNRF2 and pAkt. These observations were reversed following pharmacological and genetic inhibition of NRF2 with bexarotene and siRNA, respectively across all the cell lines but with different levels of response, perhaps due to differential levels of basal expression of these component molecules. Bexarotene is a rexinoid and known inhibitor of NRF2 [9, 10] and is Food and Drug Administration (FDA) approved for treatment of cutaneous T-cell lymphoma [58, 59].

There is increasing interest in and use of molecularly targeted inhibitors in cancer treatment, however the efficacy of these drugs is sometimes markedly reduced due to the development of resistance. Anticancer therapeutic strategies including chemotherapy can depend on ROS manipulation to induce cytotoxicity in cancer cells. In this study, we have shown pharmacological activation of NRF2 by tBHQ, in turn increasing HER4 receptor at transcriptional and translational levels and increasing the survival of the cancer cells by reducing the cytotoxicity of the HER targeting chemotherapeutic drugs, LAP and ERL. Pharmacological and genetic inhibition of NRF2 with bexarotene and siRNA, respectively, lowered the levels of HER4 at both translational and transcriptional levels. Importantly, we have previously reported that bexarotene-dependent inhibition of NRF2 lowered expression of HER2 and HER3 receptors and enhanced the cytotoxicity of ERL and LAP in ovarian cancer cells [40].

It is contextually relevant to highlight the observation that HER4 expression can be at a high-level in cisplatin resistant ovarian cancer models [33, 34]. Increased NRF2 levels, its activation and function have been implicated in the mechanisms of resistance to cisplatin [60, 61]. Furthermore, HER4 expression appeared to correlate with chemotherapy resistant ovarian serous carcinoma and shortened overall survival of patients [34]. Interestingly, HER4 is becoming recognized as a good prognostic marker for breast cancer, especially for estrogen receptor (ER) positive and triple negative breast cancer (TNBC) [25, 28, 29, 32]. More specifically the favorable impact of HER4 expression was demonstrated in the observed prolonging of overall outcome and survival in trastuzumab-treated patients [22, 62]. In HER2 positive breast cancer, trastuzumab treatment and its acquired resistance induced HER4 upregulation, cleavage and nuclear translocation [22, 62]. A balance between levels of expression and spatial distribution of HER4 may define sensitivity and resistance to trastuzumab, and perhaps other HER targeting therapeutics like LAP and ERL, which may also support and strengthen our observations and results of this study. It has been demonstrated that HER4 can be upregulated in LAP-resistant breast cancer cells and while ablation of HER4 led to apoptosis, this was not the case for HER1-3 [31]. Similar data were obtained for the response to trastuzumab. This led to the suggestion that HER4 may have a key role in HER2-positive breast cancer cells resistant to HER2 inhibitors while its role in sensitive cells is minimal [31]. HER4 appeared to modulate the PI3K/AKT pathway to confer the resistant phenotype. This is consistent with our observation that pharmacological or genetic knockdown of NRF2 reduces both HER4 and pAkt levels, and pharmacological activation of NRF2 induces both HER4 and pAkt and confers protection and survival of cancer cells following LAP and/or ERL treatments. In addition, the findings support the concept that NRF2 status and function in the cell could be a contributing determinant and basis of cellular sensitivity and response (susceptibility and resistance) of some cancers to LAP and/or ERL.

Next, we sought evidence that NRF2 controls HER4 expression in various *in vitro* and *in vivo* cancer models. We did this by performing bioinformatic analysis of microarray data of ovarian cancer cells, NSCLC cells, rat hepatocellular carcinoma tissues and NSCLC patient tissues [44]. In our analysis we focused on the NRF2 network using a knowledge based approach as before [63]. Generally, we could demonstrate that NRF2 and HER4 follow similar expression patterns along with other NRF2 controlled antioxidant response pathway genes in both the *in vitro* and the *in vivo* models (Figure 4). Significant changes in gene expression of NRF2 and its network signalling were observed after small molecule RTKi treatment with LAP and/or ERL. The observed modulation (upregulation) of NRF2 by HDAC inhibitor YF45A alone and the reversal with the combination with ERL highlighted the notion that epigenetics may contribute to the modulation of NRF2 function and the response to small molecule RTKi treatment with ERL. This by extension complements our previous reports suggesting the role of epigenetics in the inhibition of NRF2 function by HER2-targeted antibody therapy [41].

Finally, we developed a cell line with partial resistance to ERL which also had cross-resistance to LAP. This cell line had decreased constitutive ROS and increased constitutive total glutathione, HER4, pNRF2, pAkt and NRF2-dependent HO-1 relative to wild-type PEO1 cells. These observations are consistent with NRF2 activation leading to induction of HER4, cellular protection, and resistance to ERL and LAP. These results extend and confirm the role of NRF2 in modulating the cytotoxic effect of ERL and LAP [40]. This study expands the role of NRF2 as a key element in regulating the HER family receptors, their expression and function, and in influencing the sensitivity and response to HER targeting therapies. Furthermore, these findings provide support for the existence of a direct link and interaction between ROS, HER4 expression and NRF2 antioxidant response pathway.

In conclusion, this study has provided evidence for a novel role of NRF2 in regulating the HER4 receptor and has exemplified cross talk between ROS, NRF2 and HER4 receptor. Using bioinformatic analysis, we present evidence that NRF2 and HER4 expression follow a similar pattern in various *in vitro* and *in vivo* models. Furthermore, our recent studies on the regulation of HER1, HER2 and HER3 by NRF2 to oppose HER targeted antibody therapy and chemotherapy [40, 41, 63], support the assertion that NRF2 also regulates HER4 receptor function. Results from this study showed that the pharmacological activation of NRF2 attenuates the cytotoxicity of HER targeting small molecules such as ERL and LAP. It is evident that NRF2 regulates HER4 receptor levels and function and may likely be contributing resistance to ERL and perhaps LAP in ovarian cancer cells. Clinical trials to date have demonstrated only very limited activity of ERL and LAP in ovarian cancer. A phase II trial of ERL as monotherapy indicated a 6% response rate and 44% disease stabilization rate [64] and combination strategies with chemotherapy or bevacizumab have not yet proven effective [65]. For LAP, while monotherapy appears ineffective there has been interesting in its combination with carboplatin and paclitaxel [66]. Our current findings suggest that preclinical studies with NRF2 inhibitors in combination with HER inhibitors would be of interest. If effective modulation of the ROS/NRF2/HER4 axis would be a novel strategy to improve the efficacy of chemotherapeutic drugs by sensitization or by overcoming drug resistance.

## Abbreviations

AKT: serine-threonine kinase
ANOVA: analysis of variance
AREs: antioxidant response elements
EGFR: epidermal growth factor receptor
ERL: erlotinib
FBS: fetal bovine serum
FDR: false discovery rate
GEO: Gene Expression Omnibus
GSH: glutathione
HRG: heregulin
HDAC: histone deacetylase
HER4: human epidermal growth factor receptor 4
HO-1: heme oxygenase-1
LAP: lapatinib
Maf: macrophage activating factor
NRF2: nuclear factor erythroid 2-related factor 2
NSCLC: non-small cell lung cancer
pNRF2: phosphorylated-nuclear factor erythroid 2-related factor
ROS: reactive oxygen species
RTKi: receptor tyrosine kinase inhibitor
SAM: significance analysis of microarrays SD: standard deviation
siRNA: small interfering RNA
STAT: signal transducers and activators of transcription
tBHQ: tert-butylhydroquinone
TKIs: tyrosine kinase inhibitors UT: untreated

## Declarations

### Copyright

© The Author(s) 2021.
